# Downhill running impairs peripheral but not central neuromuscular indices in elbow flexor muscles

**DOI:** 10.1016/j.smhs.2021.03.001

**Published:** 2021-03-23

**Authors:** Xin Ye, Robert J. Benton, William M. Miller, Sunggun Jeon, Jun Seob Song

**Affiliations:** a Department of Rehabilitation Sciences, University of Hartford, West Hartford, CT, USA; b Department of Health, Exercise Science, and Recreation Management, University of Mississippi, University, MS, USA; c School of Kinesiology, Applied Health and Recreation, Oklahoma State University, Stillwater, OK, USA

**Keywords:** Muscle damage, Twitch interpolation, Voluntary activation, Surface EMG, Non-local effect

## Abstract

The purpose of this study was to examine the effects of a 1-h downhill running exercise on the elbow flexor muscles’ neuromuscular functions. Seventeen adults (Control [CON]: *n* = 9; Experimental [EXP]: *n* = 8) completed this study. The CON rested for 30 min while the EXP performed the downhill running. Before, 10 min, 24 h, and 48 h after the interventions, dependent variables (knee extensor muscle soreness, elbow flexion and knee extension isometric strength, elbow flexion resting twitch and voluntary activation [VA], and the biceps surface electromyography [EMG] amplitude) were measured. Knee extensor muscle soreness was significantly greater in the EXP than the CON group following the intervention throughout the entire 48 h. This was accompanied by the greater decline in the knee extension strength in the EXP than the CON (*mean* ± *SD*: -6.9 ± 3.4% vs. 1.0 ± 3.2%, *p* = 0.044). The elbow flexion strength, VA, and EMG amplitude were not affected by the exercise. However, the decline of the elbow flexion resting twitch was greater in the EXP than the CON (−19.6 ± 6.3% vs. 8.7 ± 5.9%, *p* = 0.003). Therefore, the downhill running impaired the remote elbow flexor muscles at a peripheral level.

## Introduction

Downhill running is a common component in a long-distance race with varied elevations (e.g., St. George Marathon in St. George, Utah, USA). Different from running on a flat surface, running downhill imposes unique biomechanical demands to some lower limb muscles, such as knee extensors, ankle flexors, and hip extensors.^1^ The main roles of these muscles during normal gait are to support the body weight against gravity and to absorb shock. During downhill running, however, the significantly increased change in the knee angle between foot strike and peak flexion angle^2^ indicates that the knee extensor muscles are actively lengthened to a greater degree than in level running. Thus, this biomechanical difference can result in more eccentric work for the knee extensors at their longer length, which can induce greater muscle damage in downhill than level or uphill running.^3^ For example, after downhill running exercises, muscle damage markers such as the knee extension strength and the muscle soreness have been found to increase and remain elevated for days.^4^^,^^5^ Anecdotally, this is usually reflected by a longer-than-normal recovery time following a long-distance race that contains a downhill running portion, when compared to the level or uphill running with the same running distance or intensity.

Skeletal muscle damage is referred to as a state of prolonged impaired muscle functions resulting from physical damage to muscle tissues.^6^ In the exercise and sports field, physical activities that contain unaccustomed intense skeletal muscle lengthening (eccentric) contractions can often induce muscle damage. Evidence suggests that exercise-induced muscle damage (EIMD) can lead to the decreased activation of the injured muscle from the central nervous system,^7^, ^8^, ^9^, ^10^ in addition to the reduced intrinsic force-producing capabilities of the injured muscle fibers.^11^^,^^12^ As one of the central fatigue indices, voluntary activation (VA) has been shown to decrease immediately after eccentric knee extension exercise but recovered within an hour.^13^ In addition, motor unit firing properties can also be altered immediately after a bout of eccentric exercise,^14^, ^15^, ^16^ as compared to the concentric or isometric exercise. These changes in the nervous system may be linked to the different neural activation strategies during the concentric vs. eccentric contractions.^17^

In addition to influencing the intervened muscle groups, high-intensity exercise interventions can also affect remote non-exercised or less active muscle groups. These phenomena have been reported, and they were generally described as the term non-local muscle fatigue.^18^ For example, fatiguing exercises can induce muscle performance impairments in non-exercised muscle groups which are located contralateral, ipsilateral, inferior, or superior to the exercised muscle groups.^18^^,^^19^ Briefly, this non-local fatiguing effect was likely due to the fatiguing exercise-induced central fatigue, reflected by the decreased VA.^18^^,^^20^ It is important to point out though, the majority of the non-local research studies examine contralateral homologous muscles (focusing on the contralateral crossover effect), with fewer examining the heterologous muscles. Additionally, the majority of the studies investigating non-local muscle fatigue were also limited to the examinations of the pre-vs. immediate post-measurements, rather than the potential prolonged effects, which may last for hours or days after high-intensity exercise. This topic can be interesting and practically important: if a prolonged non-local impairment effect were positive, then exercise performance of a body part (e.g., upper limb muscles) can possibly be impaired even on the subsequent days following the high-intensity exercise of a different body part (e.g., lower limb muscles).

Even though non-local effect research has been emerging over the last decade, very few non-local research studies have used eccentric contractions as the exercise intervention, which can induce prolonged neuromuscular functions and performance declines. Recently, evidence has shown that high intensity unilateral eccentric knee extension exercise can contribute to the reduced electromyographic (EMG) amplitude and physical work capacity of the non-exercised contralateral muscle.^21^ Specifically, the contralateral knee extension maximal strength and submaximal sustained contraction task failure time were significantly reduced immediately after the unilateral eccentric exercise protocol, and remained low for the following 2 days. The authors proposed that the eccentric exercise-induced delayed-onset muscle soreness (DOMS) could be the source influencing the non-exercised contralateral side. Briefly, the perception of pain (due to the exercise-induced muscle soreness) within the damaged quadriceps muscle may alter cerebral motor plans,^22^ thereby reducing the muscle performance of the contralateral non-exercised homologous muscle. If this were the case, then it is possible that a damaging eccentric exercise-induced prolonged muscle soreness and pain may even have a long-lasting non-local effect on the muscles that are located away from the exercised body parts.

Therefore, in this study, we utilized the 1-h downhill running protocol to induce lower limb muscle damage, and the main purpose was to determine if this damaging protocol would cause prolonged declines in the remote heterologous upper limb muscles (elbow flexors) neuromuscular functions and performance. In addition, surface EMG was used to examine the muscle excitation levels of the biceps brachii when performing submaximal contractions (e.g., 50% of the maximal isometric force), as examined in Hedayatpour et al.^21^ It is important to mention that this may not belong to the traditional “non-local effect” research, as downhill running is a whole-body exercise, where upper limb muscles are active during the run, even though they are not as intensely exercised as the lower limb muscles. It was hypothesized that the 1-h downhill running exercise may induce a prolonged performance impairment (e.g., strength loss) in the remote upper limb muscles. It is also important to emphasize the unique characteristic of the current study: very few downhill running studies have focused on the remote upper limb muscles’ responses following a prolonged bout of downhill runs. The information obtained from this study regarding the potential prolonged remote effects following downhill running EIMD can be important for exercise and sports professions when developing training and/or recovery strategies for individuals who compete in long-distance races containing a significant amount of downhill running.

## Material and methods

### Study design

**Fig. 1:**
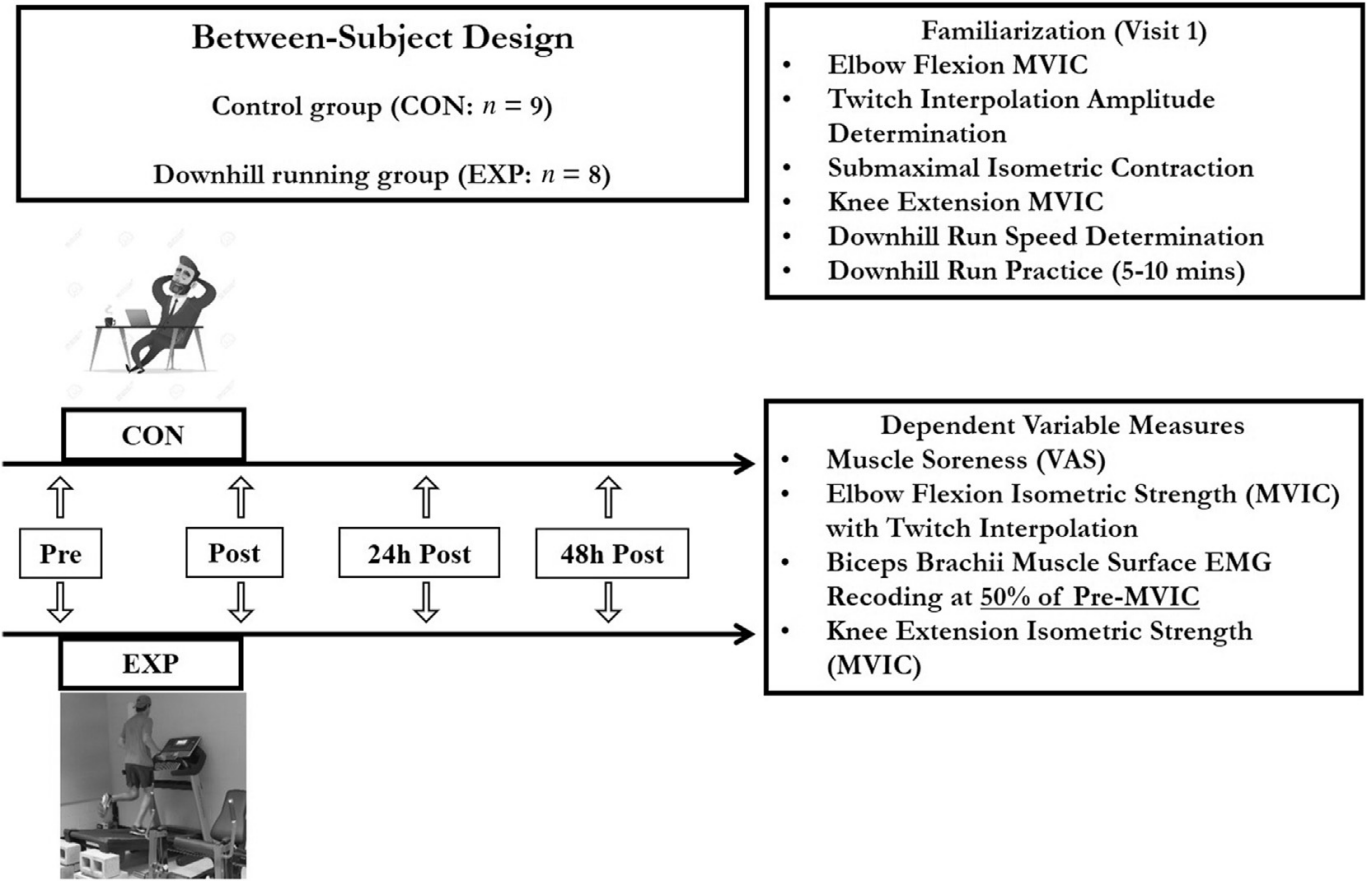
Experimental design and testing procedures of this study.

To examine whether a 1-h downhill running exercise would cause prolonged declines in the neuromuscular parameters (e.g., isometric strength, VA, muscle excitation) of the non-local upper limb elbow flexor muscles, a between-group (experimental [EXP] vs control group [CON]) repeated-measures design was utilized (Fig. 1). Subjects assigned to the EXP group completed a 1-h downhill running exercise session. Subjects assigned to the CON group sat and rested for 30 min. Before (Pre), 10 min after (Post), 24 h (Post24), and 48 h (Post48) after the downhill running exercise or CON, dependent variables (knee extensor muscle soreness, elbow flexion isometric strength, elbow flexion resting twitch and VA, surface EMG amplitude of the biceps brachii muscle during submaximal contractions, and knee extension isometric strength) were measured. All tests were done on the dominant arms and legs, based on the subjects’ throwing and kicking preferences, respectively.

### Subjects

A total of 17 healthy and physically-active adults (2 females, both were in the EXP group) participated in and completed this study (EXP group: *n* = 8, Age = 20.5 ± 1.4 years, Height = 175.1 ± 6.2 cm, Weight = 67.6 ± 3.2 kg; CON group: *n* = 9, Age = 21.0 ± 1.1 years, Height = 180.6 ± 6.3 cm, Weight = 78.2 ± 10.9 kg). On average, the subjects engaged in aerobic exercises (mostly jogging and running) 4 h per week prior to the study. None of the subjects had previous experience with downhill running. Before participation, written consent was obtained from each subject via the consent form. All subjects also completed a pre-exercise questionnaire, which indicated that they had no current or previous neuromuscular and musculoskeletal disorders, and they were healthy to engage in exercise. In addition, all subjects were instructed to refrain from any exercises or running activities during the entire study period and to maintain all their normal routines, such as dietary intake, hydration, and sleep for the duration of their participation. This study was approved by the University of Mississippi Institutional Review Board (approval code: 19-106). We estimated the sample size based on the data from a previous study with a similar design,^23^ where the authors examined the prolonged downhill running on of elbow flexor muscles’ strength and neuromuscular indices. Based on the observed large effect size from Branderberg et al.,^23^ it was shown that at least 6 subjects per group were necessary for the comparisons between the EXP and the CON, with *f* = 0.40, the alpha level of 0.05, and power (1−*β*) of 0.80 ^24^ by G∗Power (G∗Power 3.1.9.4, Heinrich-Heine-Universitat Dusseldorf, Dusseldorf, Germany).

### Procedures

#### Familiarization Visit

**Fig. 2:**
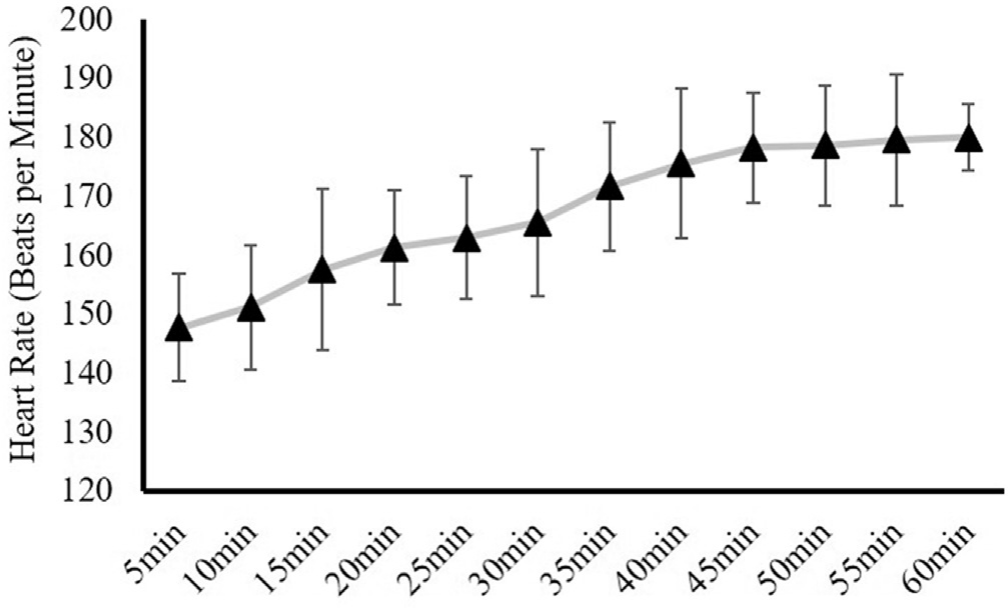
Experimental group (EXP) participants' heart rate (*Mean* ± *SD*) at 5, 10, 15, 20, 25, 30, 35, 40, 45, 50, 55, and 60 min of the 1-h downhill running exercise.

The first visit to the laboratory was to familiarize the subjects with the testing procedures and the downhill running exercise (only EXP group). During this visit, the subjects' standing height and body mass were measured first, followed by the familiarization to the Visual Analog Scale (VAS) muscle soreness measurement. Then the subjects were familiarized with the elbow flexion isometric strength testing. Specifically, they practiced contracting their dominant elbow flexor muscles against an immovable apparatus (Fig. 2a) several times with 50% of the maximal effort as the warm-up, and then followed by two maximal voluntary isometric contractions (MVICs). Following this practice, the researcher then proceeded to clean the subjects’ bicep brachii muscle belly with alcohol wipes, and then use a razor to shave the surface hair and the dead skin. Once the skin was prepared, two stimulating electrodes (5 × 5 cm square electrodes, model USX2020, Axelgaard Manufacturing Co., Ltd., Fallbrook, CA, USA) were placed on the skin over the proximal belly (cathode) and the distal tendon (anode) of the biceps brachii muscle based on the setup from Magnus et al.^25^ The stimulating electrodes were connected to a constant-current stimulator (Digitimer model DS7AH; Hertfordshire, England, UK). With the exact same setup as the isometric strength testing, the subjects were asked to simply relax their elbow flexor muscles for the stimulation amplitude determination. This involved the researchers starting with a series of stimuli (paired pulses at 100 Hz, 200 μs pulse-width) at 60 milliamps (mA), and increasing by 20 mA every 20 s until the involuntary resting elbow flexion twitch force reached a plateau and then demonstrated a decline on 2 consecutive stimulations. The exact locations of the stimulating electrodes were marked with a permanent marker pen.

After the stimulation amplitude determination, the subject was familiarized with the submaximal isometric contractions at the 50% MVIC. Once completed, the subjects were asked to rest as the researcher set up the leg extension machine (Steelflex PLLE 200; Steelflex Fitness, Taipei, Taiwan China) for the familiarization of the dominant knee extension isometric strength testing. This familiarization followed a similar protocol as used in the elbow flexion isometric strength testing (e.g., several submaximal knee extension contractions followed by two MVICs). Finally, as for subjects in the EXP group, they were familiarized with the downhill running protocol. Specifically, the subjects’ maximal heart rate (HR) was estimated by using the Fox formula^26^: HR_max_ = 220 - age. Then the subjects ran with an HR monitor (Polar H7 Bluetooth Heart Rate Sensor, Polar Electro Inc., Bethpage, NY, USA) for a duration of 5–10 min on a treadmill set at 0% grade at a pace corresponding to 85% of their HR_max_. After the pace was determined, the subjects were told to take a rest, during which the treadmill was adjusted to the downhill setup at −10% grade. The subjects were instructed to run at the determined pace for a few minutes to ensure they would be comfortable with this exercise. Once the familiarization was completed, the subjects were asked to return to the laboratory for the remaining experimental visits for any three consecutive days at roughly the same time each visit.

#### Experimental visits

During the first experimental testing visit (Visit 2), the subjects returned to the laboratory where Pre-testing dependent variables were measured in the following order: knee extensor muscle soreness, elbow flexion isometric strength with twitch interpolation, surface EMG amplitude during the submaximal elbow flexion isometric contractions, and knee extensor isometric strength. If assigned to the EXP group, following the Pre-tests, the subjects were allowed to warm up for approximately 5 min with the running speed they preferred, before they attempted to complete the 1-h downhill running exercise with the pre-determined pace (*mean* ± *SD* = 7.7 ± 0.7 miles per hour). During the run, we did not specifically instruct the subjects how to adjust the upper and lower body gait patterns (e.g., leg and arm swings). Instead, every subject adjusted their postures to their comfortable positions throughout the whole running intervention. The Subject's HR was monitored every 5 min throughout the entire downhill running protocol (Fig. 2). Upon completion, subjects were given 10 min to rest and rehydrate before the Post-measurements. For the CON group, instead of the 1-h downhill running, the subjects sat and rested for 30 min. Ten minutes after, 24 h, and 48 h following the EXP or CON, the same dependent variables were assessed in the exact same manner and order as they were measured during the Pre-testing.

### Measurements

#### Knee extensor muscle soreness

The muscle soreness was assessed using a 100-mm visual analog scale (VAS).^27^ The VAS scale shows “No soreness” on the far-left side and “Unbearable pain” on the far-right side. Subjects were asked to fully flex and extend their dominant quadriceps muscles several times and to mark a vertical line on the VAS scale at the location representing their perceived soreness level from the knee extensor muscles.

#### Elbow flexion isometric strength with twitch interpolation

**Fig. 3:**
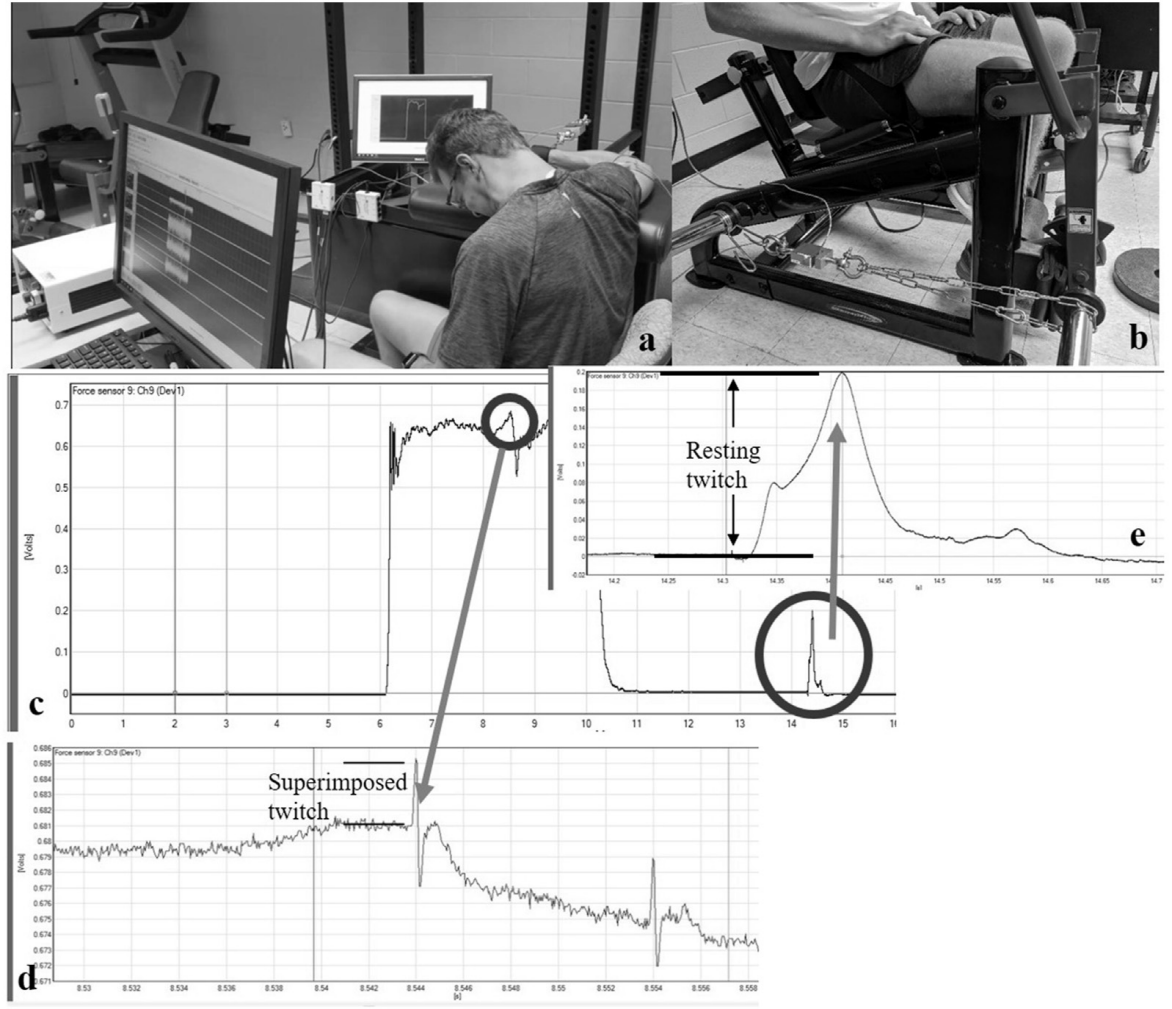
a. Unilateral elbow flexion maximal isometric contraction and twitch interpolation setup; b. Unilateral knee extension maximal isometric strength testing setup; c. An example of the force data of the elbow flexion maximal isometric contraction with twitch interpolation applied; d. The measurement of superimposed twitch force; e. The measurement of resting twitch force.

For the elbow flexion isometric strength testing, the subjects were seated with an upright position, with the dominant arm resting on a padded table, where the pad was firmly pushing against the subjects' armpit. Meanwhile, the subject's dominant wrist was attached by a strap, which was connected to a load cell (Model SSM-AJ-500; Interface, Scottsdale, AZ, USA). The other end of the load cell was connected to an immovable steel frame. Extra care was taken to ensure the subjects' arm was parallel to the floor, and the elbow joint was at a 90-degree angle (Fig. 3a). Following the brief warm-up, the subjects were asked to produce three trials, 3-s maximal isometric elbow flexions with a 60-s rest between trials as fast as they could, and then as hard as possible in order to produce maximal explosive force. For each MVIC, the researchers counted down from 3 and then verbally encouraged the subjects with a “pull, pull, pull” until it was time for them to relax. During the 2nd and 3rd MVICs, the twitch interpolation technique was performed. This technique was first used by Merton,^28^ and reported to be valid and highly reliable in measuring the voluntary activation level of a muscle.^29^ With the same stimulation electrodes placements as during the Familiarization Visit, a paired pulse stimulus (100 Hz, 200 μs pulse-width) was delivered at about 2.5 s into the MVIC, followed by the same paired pulse stimulus delivered about 4 s after the MVIC, during the fully relaxed state. The stimulation current intensity (range = 78–139 mA) was set at 130% of the current intensity recorded from the amplitude determination procedure during the Familiarization Visit.

#### Elbow flexion submaximal isometric contractions

To monitor neuromuscular efficiency throughout the entire experiment, the submaximal isometric contraction testing was performed with the same setup as the elbow flexion isometric strength testing. The subjects contracted their elbow flexor muscle with a “ramp-up, hold, and ramp-down” (i.e., trapezoid) manner. A computer monitor was provided to display the target force template, as well as the subject's real-time force output. Specifically, the subjects gradually increased the force output from 0% (rest) to 50% of their Pre-MVIC for 5 s (10% MVIC per second), held it for 10 s, and then gradually decreased it to 0% MVIC for 5 s. Two repetitions of the trapezoid contraction were performed at each measurement point. In addition, the force template (50% of the Pre-MVIC from Visit 2) was always constant throughout all experimental visits. Therefore, if there were any fatiguing effect on the elbow flexor muscles due to the downhill running, then an increased normalized EMG amplitude should be seen.

### Knee extension isometric strength

For the knee extension isometric strength testing, the subjects performed three trials, 3-s MVICs with a 60-s rest between trials. The same load cell (Model SSM-AJ-500; Interface, Scottsdale, AZ, USA) was used to measure the isometric force generated by the knee extensor muscles. Specifically, one end of the load cell was connected to the lever of the ankle pad, and the other end was connected to the backside of the knee extension machine through a steel chain (Fig. 3b). Prior to any contraction, the knee extension machine was adjusted so that the subject's back was upright, and the knee joint was approximately 75° (full knee extension = 180°). The ankle pad was also adjusted based on the subjects' preference. Throughout the entire study, the ankle pad and back positions were recorded by the researchers to ensure that these positions were consistent. The subjects were strapped in with a Velcro® belt around the waist level to minimize hip movements. Additionally, both of their arms were rest and placed on top of their chests during the maximal contractions. Prior to the maximal contractions, the subjects were instructed to perform several warm-up contractions with 50% of their maximal effort. For each MVIC, the researchers counted down from 3 and then verbally encouraged the subjects with a “push, push, push” until it was time for them to relax.

### Data analyses

#### Force

After the elbow flexion and knee extension isometric strength testing, the offline force signals were digitized with a 16-bit analog-to-digital converter (NI USB-6259 M Series; National Instruments, Austin, TX, USA), sampled at 20 kHz with a 16-channel Bagnoli desktop EMG system (Delsys, Inc., Natick, MA, USA), and then stored in a laboratory computer (Dell XPS 8900, Round Rock, TX, USA) for further analyses. For each 3-s MVIC, the peak 0.5 s window (rather the superimposed twitch area) was chosen and then calculated as the maximal force output. The average of the three maximal force outputs was then calculated as the maximal isometric strength.

#### Voluntary activation (VA)

During the elbow flexion isometric strength testing, the superimposed twitch force and resting twitch force were first selected and calculated for each MVIC (Fig. 3c–e). The averaged (between the 2nd and 3rd MVIC) twitch values were then used to calculate the VA using the following equation: VA (%) = (1 - superimposed twitch force/resting twitch force) × 100%.^30^

#### Surface EMG acquisition and signal processing

During the elbow flexion maximal isometric contractions, as well as the submaximal trapezoid contractions, bipolar surface EMG signals were recorded through an EMG sensor (dEMG sensor, Delsys, Inc., Natick, MA) attached to the biceps brachii muscle belly based on the sensor location recommendations from SENIAM.^31^ This sensor array comprised five cylindrical probes (0.5 mm diameter) located at the center and the corners of a 5 × 5 mm square. Thus, 4 separate bipolar EMG signals were detected based on the pairwise differences in the five probes, and all 4 channels were selected for subsequent analyses. The reference electrode (USX2000; Axelgaard, Fallbrook, CA, USA) was placed on the seventh cervical vertebrae (C7). Prior to any electrode placements, the researcher shaved and cleaned the skin surface with rubbing alcohol, and medical tapes were used to firmly fixate the electrodes on the skin sites. All analog bipolar EMG signals were collected and amplified (gain = 1000) with a Bagnoli 16-channel EMG system (Delsys, Inc., Natick, MA, USA) and filtered with high and low pass filters set at 20 Hz and 450 Hz, respectively. The filtered signals were then digitized at a sampling rate of 20 kHz with a 16-bit analog-to-digital converter (NI USB-6259 M Series; National Instruments, Austin, TX). Synchronized with the maximal force signal (highest 0.5-s portion from the MVICs), the amplitude of the selected EMG signal from each channel was calculated as the root-mean-square (RMS), and the EMG amplitude of the biceps brachii was then calculated as the average of the RMS values from the 4 EMG channels. This maximal EMG amplitude value then served as a normalization value for each recorded EMG signal from the submaximal isometric contractions. Specifically, the EMG signal from the mid-6-second plateau region of the submaximal isometric trapezoid contraction was selected, and its RMS value was normalized as a percentage of the maximal EMG amplitude.

### Statistical analyses

Assumptions for normality of distribution for these dependent variables were checked and confirmed using the Shapiro-Wilk test. The baseline (Pre-values during the second visit) dependent variables were examined via the independent *t*-tests between groups [CON vs. EXP]. The absolute change values (Δ: Post-Pre; Post24-Pre; Post48-Pre) of the VAS, VA, and the normalized EMG amplitude during the submaximal isometric trapezoid contractions, along with percent change values (%Δ: Post-Pre; Post24-Pre; Post48-Pre) of the elbow flexion and knee extension isometric strength, and elbow flexion resting twitch force, were calculated for further statistical analyses. Separate two-way (time [ΔPost-Pre vs. ΔPost24-Pre vs. ΔPost48-Pre] × group [CON vs. EXP]) mixed factorial analyses of variance (ANOVAs) tests were conducted to examine the change scores of all dependent variables across time between groups. When appropriate, the follow-up tests included one-way repeated-measures ANOVA with Bonferroni-adjusted pairwise comparisons, as well as independent *t*-tests. The partial $\eta^{2}$ statistic was provided for all repeated measure comparisons, with values of 0.01, 0.06, and 0.14 corresponding to small, medium, and large effect sizes, respectively.^24^ All statistical tests were conducted using statistical software (IBM SPSS Statistics 25.0; IBM, Armonk, NY) with an alpha set at 0.05. The data were presented as *mean* ± *standard deviation* (*SD*).

## Results

### Baseline values

Table 1 shows the baseline values for dependent variables for both EXP and CON groups. No significant differences were observed for all dependent variables.

**Table 1:** Baseline (Pre-testing) comparisons of the dependent variables (mean ± standard deviation) for both experimental (EXP) and control (CON) groups.

Variable	EXP (*n* = 8)	CON (*n* = 9)	*P*-value
**VAS (mm)**	4.2 ± 4.6	17.7 ± 21.3	0.101
**Elbow Flexion Isometric Strength (N)**	257.2 ± 62.1	293.0 ± 48.6	0.203
**Knee Extension Isometric Strength (N)**	372.2 ± 102.3	441.0 ± 121.4	0.229
**Resting Twitch (N)**	35.9 ± 22.3	47.3 ± 18.3	0.264
**Voluntary Activation (%)**	93.27 ± 4.63	94.59 ± 3.58	0.615
**Normalized EMG Amplitude (%)**	51.35 ± 14.60	45.16 ± 12.69	0.364

### Change (Δ) of knee extensor muscle soreness

**Fig. 4:**
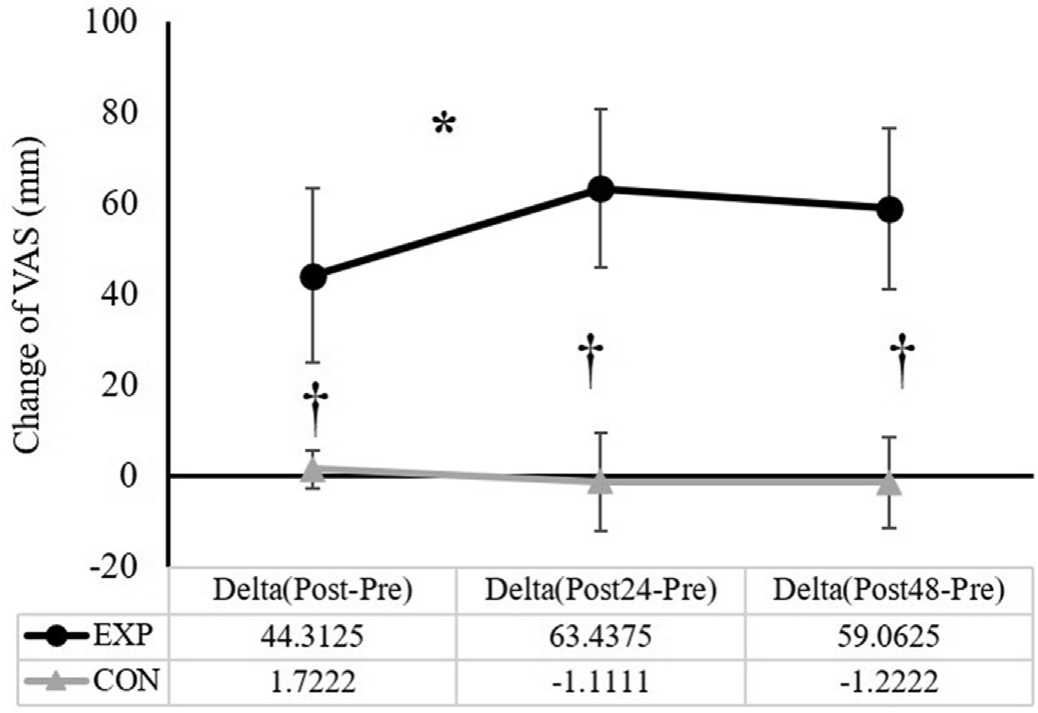
The comparisons of the change (Δ) in knee extensor muscle soreness (*mean* ± *SD*) between the experimental (EXP) and control (CON) groups. **∗** Significant difference between Δ(Post-Pre) and Δ(Post24-Pre) **†** Significant difference between EXP and CON.

The two-way ANOVA for the ΔVAS indicated a significant group × time (*F*_(2,30)_ = 5.873, *p* = 0.007, partial $\eta^{2}$ = 0.281) interaction, and a main effect for group (*F*_(1,30)_ = 101.058, *p* < 0.001, partial $\eta^{2}$ = 0.871). The follow-up one-way repeated measure ANOVAs showed a significant main effect for time for the EXP group (*F*_(2,14)_ = 4.814, *p* = 0.026, partial $\eta^{2}$ = 0.407), but not for the CON (*F*_(2,16)_ = 0.672, *p* = 0.439, partial $\eta^{2}$ = 0.077). For the EXP, the Bonferroni-adjusted pairwise comparisons indicated a significantly smaller VAS value at Δ(Post-Pre) than that at Δ(Post24-Pre) (*mean* ± *SD*: 44.3 ± 19.3 mm vs. 63.4 ± 17.5 mm, *p* = 0.002). In addition, the independent *t*-tests showed significant differences between the two groups at all time points (Fig. 4).

### Percent change (Δ%) of knee Extension and elbow flexion isometric strength

For the knee extension isometric strength, the two-way ANOVA showed that there was no significant group × time (*F*_(2,30)_ = 2.153, *p* = 0.134, partial $\eta^{2}$ = 0.126) interaction. However, there were main effects for group (*F*_(1,30)_ = 2.929, *p* = 0.044, partial $\eta^{2}$ = 0.163) and time (*F*_(2,30)_ = 9.011, *p* = 0.001, partial $\eta^{2}$ = 0.375). With all time points merged, the follow-up independent *t*-test showed a significant difference between the two groups (EXP vs. CON = −6.9 ± 3.4% vs. 1.0 ± 3.2%, *p* = 0.044). With both groups merged, significantly greater percent declines of the knee extension isometric strength were observed at Δ(Post-Pre) than that at Δ(Post24-Pre) (*mean* ± *SD*: -10.1 ± 2.2% vs. −2.2 ± 2.8%, *p* = 0.014), and at Δ(Post-Pre) than that at Δ(Post48-Pre) (*mean* ± *SD*: -10.1 ± 2.2% vs. 3.5 ± 3.7%, *p* = 0.004).

The two-way ANOVA for the elbow flexion isometric strength showed that there was no significant group × time (*F*_(2,30)_ = 0.317, *p* = 0.731, partial $\eta^{2}$ = 0.021) interaction, nor a main effect for group (*F*_(1,30)_ = 0.236, *p* = 0.634, partial $\eta^{2}$ = 0.016). However, there was a main effect for time (*F*_(2,30)_ = 5.423, *p* = 0.010, partial $\eta^{2}$ = 0.266). With two groups merged, significantly greater percent declines of the elbow flexion isometric strength were observed at Δ(Post-Pre) than that at Δ(Post24-Pre) (*mean* ± *SD*: -7.0 ± 2.1% vs. −1.8 ± 1.9%, *p* = 0.003), and at Δ(Post-Pre) than that at Δ(Post48-Pre) (*mean* ± *SD*: -7.0 ± 2.1% vs. −1.8 ± 2.0%, *p* = 0.038).

### Change (Δ) of elbow flexion resting twitch and voluntary activation (VA)

**Fig. 5:**
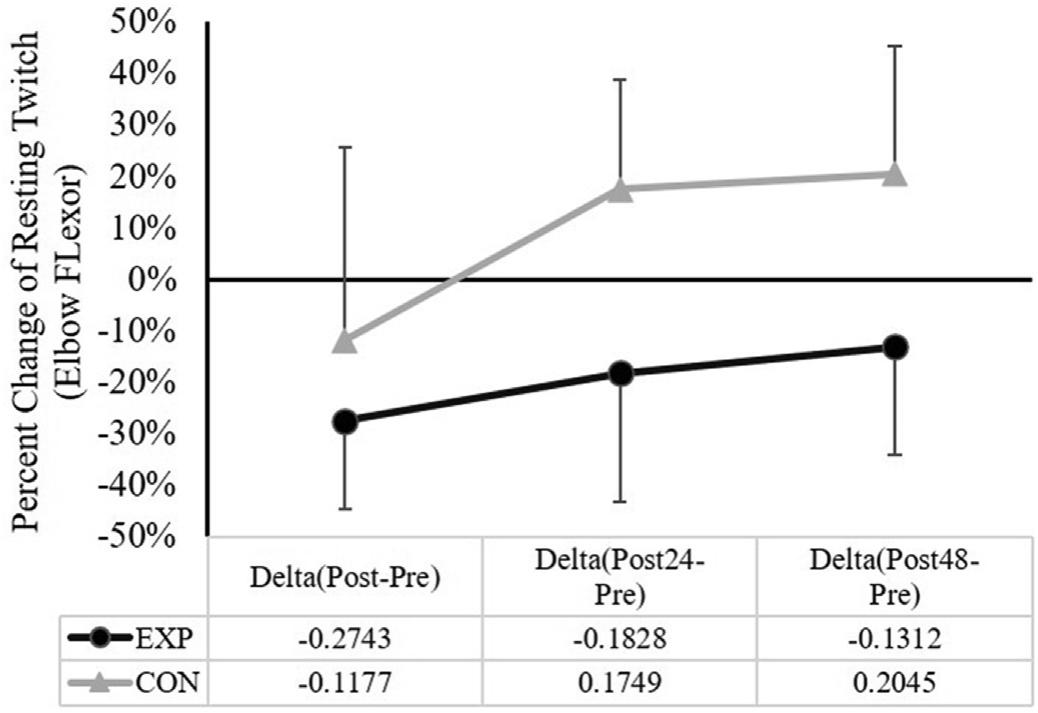
The comparisons of the percent change (Δ) in elbow flexion resting twitch (*mean* ± *SD*) between the experimental (EXP) and control (CON) groups. Note: Significant main effect for group (time points merged EXP vs. CON = −19.6 ± 6.3% vs. 8.7 ± 5.9%, *p* = 0.003).

For the elbow flexion resting twitch force, the two-way ANOVA showed that there was no significant group × time (*F*_(2,30)_ = 1.113, *p* = 0.342, partial $\eta^{2}$ = 0.069) interaction. However, there were main effects for group (*F*_(1,30)_ = 10.760, *p* = 0.005, partial $\eta^{2}$ = 0.418) and time (*F*_(2,30)_ = 5.646, *p* = 0.008, partial $\eta^{2}$ = 0.273). With all time points merged, the follow-up independent *t*-test showed a significant difference between the EXP and CON groups (EXP vs. CON = −19.6 ± 6.3% vs. 8.7 ± 5.9%, *p* = 0.003; Fig. 5). When collapsed across groups, significantly greater percent declines of the elbow flexion resting twitch were observed at Δ(Post-Pre) than that at Δ(Post24-Pre) (*mean* ± *SD*: -19.6 ± 6.9% vs. −0.4 ± 5.7%, *p* = 0.025), and at Δ(Post-Pre) than that at Δ(Post48-Pre) (*mean* ± *SD*: -19.6 ± 6.9% vs. 3.7 ± 5.5%, *p* = 0.03).

The two-way ANOVA for the elbow flexor muscle VA showed that there was no significant group × time (*F*_(2,30)_ = 1.212, *p* = 0.320, partial $\eta^{2}$ = 0.119) interaction, nor main effects for group (*F*_(1,30)_ = 1.394, *p* = 0.268, partial $\eta^{2}$ = 0.134) and time (*F*_(2,30)_ = 1.214, *p* = 0.320, partial $\eta^{2}$ = 0.119).

### Change (Δ) of biceps brachii surface EMG amplitude

**Fig. 6:**
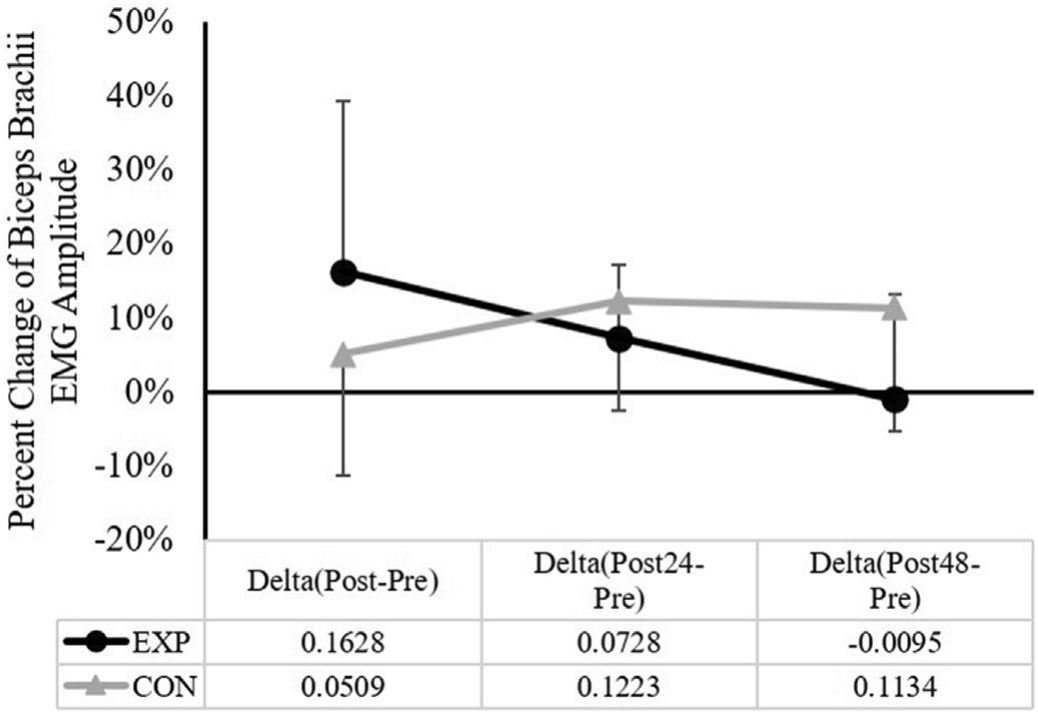
The comparisons of the change (Δ) in biceps brachii normalized EMG amplitude (*mean* ± *SD*) between the experimental (EXP) and control (CON) groups.

The two-way ANOVA for the normalized EMG amplitude during the submaximal isometric contraction indicated a significant group × time (*F*_(2,30)_ = 4.239, *p* = 0.024, partial $\eta^{2}$ = 0.220) interaction (Fig. 6). The follow-up one-way repeated measure ANOVAs showed no significant main effect for time for the EXP group (*F*_(2,14)_ = 3.176, *p* = 0.073, partial $\eta^{2}$ = 0.312) or for the CON group (*F*_(2,16)_ = 1.278, *p* = 0.305, partial $\eta^{2}$ = 0.138). In addition, the independent *t*-tests showed no significant differences between the two groups at all time points.

## Discussion

This study aimed to examine whether the 1-h of downhill running exercise could induce prolonged decrements in the neuromuscular functions (e.g., isometric strength, VA, resting twitch, and the muscle excitation during submaximal contractions) of the remote upper limb muscles such as the elbow flexors. The main findings of this study are: 1) Changes in the elbow flexion isometric strength, VA, and biceps brachii muscle excitation levels were not different between the EXP and CON; 2) The reduction in the elbow flexor muscle resting twitch was significantly greater after the downhill running exercise, relative to the CON; 3) The downhill running exercise-induced knee extensor muscle soreness were greater and remained elevated at 24 and 48 h, when compared to CON; and 4) The overall percent decline of the knee extension isometric strength following the downhill running exercise was significantly greater than that of the CON.

Lower body muscle DOMS has often been assessed following downhill running exercise interventions.^32^, ^33^, ^34^, ^35^ The exercise intervention durations across these studies ranged between 30 min and 60 min. Regardless of the different exercise duration and intensity, our study showed that the knee extensor muscle soreness increased immediately after the injury protocol (ΔPost-Pre = 44.3 ± 19.3 mm), and remained elevated for a prolonged time (ΔPost24-Pre = 63.4 ± 17.5 mm; ΔPost48-Pre = 59.1 ± 17.6 mm). Thus, the current result of the knee extensor muscle soreness is in line with the findings from these studies. Additionally, as the most noticeable effect of EMID, strength loss (as evidenced by the greater overall percent decline of the knee extension isometric strength in the EXP, relative to the CON) was also present, which indicated the damaging effect of the current downhill running exercise protocol.

With the positive indirect muscle damage markers observed from the knee extensor muscles following the current downhill running protocol, it is our main purpose to examine whether the elbow flexor muscles' neuromuscular functions and performance were altered. To the best of our knowledge, Brandenberger et al.^23^ was the only study to examine the downhill running EIMD on remote upper limb muscle functions. The authors^23^ reported the loss of the elbow flexion isometric strength following the downhill running protocol. And it was proposed that this non-local performance decrement was likely attributed to the decrements in the elbow flexor muscle VA. These findings, however, were not observed from the current study. Relative to the CON, our subjects in the EXP group did not experience significantly greater strength loss in the elbow flexor muscles. Moreover, the VA of the elbow flexor muscles was not altered. The current findings suggest that the neural factors were not affected by the 1-h downhill running exercise. It is not clear how the discrepancies between the results from the current study and those from Brandenberger et al.^23^ were originated. The slightly differed exercise protocols [e.g., downhill running speed was based on the subjects' 75% of $\dot{\text{V}}$O_2__peak_ (the highest value of oxygen uptake attained during an incremental or a high-intensity exercise test, designed to bring the participant to the limit of tolerance,^36^ where a plateau in oxygen uptake is not reached) at the flat surface in Brandenberger et al.^23^] might have contributed to the different findings. However, it is not possible to directly compare the downhill running exercise intensities between the current investigation and Brandenberger et al.^23^ throughout the 60-min of the downhill run, because heart rate data was not provided in Brandenberger et al.^23^ Another potential contributing factor could be the familiarization trial, where the participants in Brandenberger et al.^23^ did not practice the downhill run, but we had our participants practice a few minutes until they felt comfortable with this type of running exercise. Even though the downhill running practice was only a few minutes, it could have blunted the effects of the intervention and contributed to the differences in the extent of the injury in this study and that previously reported in Brandenberger et al.^23^ Besides the different downhill running protocol and the different familiarization, the subjects’ training statuses might have also played a role. For example, one of the exclusion criteria in Brandenberger et al.^23^ was running more than 10 miles per week. In the current study, however, the majority of the subjects have reached and some have exceeded this running volume prior to the participation. Perhaps our subjects were slightly more trained than those in Brandenberger et al.,^23^ leading to a dampened damaging effect^37^^,^^38^ from the 1-h downhill running exercise. This is likely supported by the evidence, that larger knee extension isometric strength percent decline (40%–50% decline across time) and larger knee extensor muscle soreness increment (80–90 mm increase in the visual analog scale) were observed in Brandenberger et al.,^23^ than those (5%–18% decline for the knee extension isometric strength; 40–60 mm increase in the 10.13039/100011439VAS) in the current experiment. In fact, when looking into the current result of the knee extension isometric strength percent decline immediately after the downhill run, our value (−18%) is close to the upper ceiling of the range (−55% to −14%) from the downhill running literature.^39^ Additionally, the isometric strength declines at 24 and 48 h after the downhill running were 5% and close to zero, respectively. This suggests a relatively less damaging effect and a faster recovery, as compared to the literature.^39^ Therefore, it is possible that the current subjects were slightly more trained than average subjects from the majority of the downhill running studies.

It is interesting that the prolonged depressions in motor performance were observed in non-exercised and less-exercised muscle groups.^21^^,^^23^ Hedayatpour et al.^21^ proposed that the eccentric exercise-induced DOMS may alter nociceptive modulations. Specifically, at the spinal level, the axons of nociceptive dorsal horn neurons cross to the contralateral side to form the anterolateral ascending tract. This ascending tract terminates at the supraspinal level, and can possibly inhibit motor cortex regions associated with the contralateral limb. This possible mechanism may explain the decreased motor performance and work capacity in the non-exercised contralateral homologous knee extensors muscles, following a unilateral eccentric knee extension damaging protocol.^21^ However, the nociceptive mechanism does not fully explain the VA decrements in the upper limb muscles observed in Brandenberger et al.,^23^ due to the unmatched patterns of changes in the elbow flexor muscle strength and the changes of pain in either the elbow flexor or knee extensor muscles in their experiment. Interestingly, in another recent experiment where knee extensor muscles’ VA was examined before and after an eccentric damaging resistance exercise protocol, the VA was only reduced immediately after the exercise intervention, but recovered within 1 h.^13^ This finding suggests that the EIMD only has a transient effect on the nervous system. Since the current VA measurement was conducted at least 10 min after the downhill running, it is possible that the VA had already been recovered in the current investigation.

The current study did have an interesting finding: the decrements of the elbow flexor muscle twitch force were significantly greater after the downhill running, as compared to those of the CON group. Since the same electrical stimulation was delivered to the biceps brachii muscle belly after the exercise protocol, a decline in the resting twitch indicates the decreased capability of the muscle fibers to generate force. Thus, this finding suggests that our downhill running protocol induced some peripheral impairments at the muscle fiber level in the remote biceps brachii muscle. It was not our intention to examine the exact mechanism(s) leading to this change, but one possibility could be the unaccustomed nature of the downhill running exercise. Specifically, with the altered posture during downhill running, the lumbar spine range of motion has been reported to be greater than that when running on level or uphill.^40^ This, along with the shift of the center of mass,^41^ are likely leading to the adjustments of the upper body posture, even though the upper limb motion was rarely examined and analyzed during downhill running exercises. Thus, it is possible that the posture adjustments during the downhill running exercise have affected the upper body posture and the arms swing motion. Although the arm muscles were not the primary movers during the downhill running, the prolonged exercise with holding an unaccustomed posture might have contributed to our result of the elbow flexor muscle resting twitch.

One question remains to be unanswered, is why the different responses of the elbow flexor isometric strength and the biceps brachii muscle resting twitch force were observed, following the downhill running protocol. Our result showed that the downhill running protocol impaired the biceps brachii muscle at the peripheral level. However, it is important to note that other synergistic muscles (e.g., brachialis, brachioradialis) and antagonist muscle (e.g., triceps brachii) that contribute to the elbow flexion force production were not examined in the current experiment. It is unknown if these muscles would have responded similarly to the biceps brachii muscle. Thus, it is possible that the non-local elbow flexor isometric strength responded differently from the biceps brachii muscle resting twitch force. Regarding the biceps brachii surface EMG (to measure neuromuscular efficiency), even though a significant interaction was found for the surface EMG amplitude during the submaximal contractions, we did not observe any further differences between the EXP and CON. However, it is important to point out, that there was a large effect size (partial $\eta^{2}$ = 0.312) for the time in the EXP group, showing a trend of decreasing surface EMG amplitude over time. Considering the resting twitch result (impairment at the peripheral level), it is likely that more neural drive was needed to sustain the force level at 50% of the maximal force level, especially at the time point right after the 1-h downhill running exercise (see Fig. 6). Thus, the EMG result suggests a trend of a temporary muscle performance decrement following the downhill running.

The current study showed some interesting findings regarding the non-local effects following a prolonged downhill running exercise. However, it is important to address some limitations of this study. First and foremost, even though the sample size of the current study was greater than the required estimation number, seventeen (CON = 9, EXP = 8) is still a relatively small sample size, specifically under a between-group research design. Second, our study contained a mix of both men and women (*n *= 2), which could have influenced the results, as previous studies have reported that the magnitude of muscle damage induced by eccentric exercise differs between men and women.^42^, ^43^, ^44^ Third, the current investigation only examined the downhill running, but not the level or uphill running protocols with the same intensity and duration. Including these running groups can help determine whether the elbow flexor muscle resting twitch response was peculiar to downhill running. Fourth, the downhill running speed determination in the current study was based on the theoretical maximal heart rate of the subjects. This, compared with the _peak_$\dot{\text{V}}$O_2_ method, might have provided a less accurate exercise intervention to induce muscle damage. Fifth, we had a fixed order of the testing measurements, which could have led to a potential order effect. Lastly, measurements such as elbow flexor muscle soreness and knee extensor VA, as well as other muscle damage blood markers such as creatine kinase were not conducted, which could potentially add a few insights to the findings of the current experiment.

## Conclusions

In conclusion, a 1-h downhill running exercise led to a prolonged increase in knee extensor muscle soreness, reduced knee extensor isometric strength, as well as the decreased upper limb elbow flexor muscles’ resting twitch force. Isometric strength and the neural factors (VA and muscle excitation) of the upper elbow flexor muscles were not altered. These findings suggest that, even though the upper limb muscles are less active during the prolonged downhill run, peripheral changes in these remote muscles can still be observed. Given that the subjects in the current study may slightly be more trained and active than average individuals, future studies should try to explore whether peripheral changes in the remote muscle groups would be magnified, if using less active or sedentary populations.

## Submission statement

The work described above has not been published previously, that it is not under consideration for publication elsewhere, that its publication is approved by all authors and tacitly or explicitly by the responsible authorities where the work was carried out, and that, if accepted, it will not be published elsewhere including electronically in the same form, in English or in any other language, without the written consent of the copyright-holder.

## Authors’ contributions

XY and RJB contributed to the design of the work; XY, RJB, WMM, SJ, and JSS contributed to the acquisition, analysis, and interpretation of data; XY, RJB, WMM, SJ, and JSS contributed to the draft of the work and revised it critically for important intellectual content; XY, RJB, WMM, SJ, and JSS approved the version to be submitted, and agreed to be accountable for all aspects of the work in ensuring that the questions related to the accuracy or integrity of any part of the work are appropriately investigated and resolved.

## Ethical approval statement

All experimental procedures in this project were in accordance with the ethical standards of 1964 Helsinki declaration and its later amendments or comparable ethical standards and were approved by the University of Mississippi Institutional Review Board (IRB Protocol #: 19-106). After each participant was given an explanation of the study, written informed consent was collected.

## Conflict of interest

This research did not receive any specific grant from funding agencies in the public, commercial, or not-for-profit sectors. There were no conflicts of interest declared from authors for the completion of this project and manuscript.
